# Comparison of Sources and Methods for the Isolation of Equine Adipose Tissue-Derived Stromal/Stem Cells and Preliminary Results on Their Reaction to Incubation with 5-Azacytidine

**DOI:** 10.3390/ani12162049

**Published:** 2022-08-11

**Authors:** Dagmar S. Trachsel, Hannah J. Stage, Sebastian Rausch, Susanne Trappe, Katharina Söllig, Gerhard Sponder, Roswitha Merle, Jörg R. Aschenbach, Heidrun Gehlen

**Affiliations:** 1 Equine Clinic: Surgery and Radiology, Department of Veterinary Medicine, Freie Universität Berlin, Oertzenweg 19b, 14163 Berlin, Germany; 2 Clinical Unit of Equine Internal Medicine, Department for Companion Animals and Horses, University of Veterinary Medicine Vienna, Veterinärplatz 1, 1210 Vienna, Austria; 3 Institute of Immunology, Department of Veterinary Medicine, Freie Universität Berlin, Robert-von-Ostertag-Str. 7, 14163 Berlin, Germany; 4 Institute of Veterinary Physiology, Department of Veterinary Medicine, Freie Universität Berlin, Oertzenweg 19b, 14163 Berlin, Germany; 5 Institute for Veterinary Epidemiology and Biostatistics, Department of Veterinary Medicine, Freie Universität Berlin, Königsweg 67, 14163 Berlin, Germany

**Keywords:** preadipocytes, mesenchymal stem cells, adipose tissue differentiation, cardiomyocyte-like cells, horse

## Abstract

**Simple Summary:**

The function of the equine heart is different from that in other species, and a species-specific in vitro model would simplify investigations in the field of equine cardiology. The recent advances in stem cell research and the availability of adipose tissue-derived stromal/stem cells (ASCs) could be a promising starting point for the development of such an in vitro model. In order to test the hypothesis that equine ASCs can be differentiated into cells resembling heart cells, we isolated ASCs from abdominal, retrobulbar, and subcutaneous adipose tissue after collagenase digestion or from direct cultivation of explants. Both techniques resulted in similar yields of cells displaying morphological, immunophenotypical, and molecular biological characteristics of mesenchymal stem cells. Abdominal adipose tissue was found to be most suitable for ASC isolation in equines. However, contrasting earlier studies performed with ASCs from other species, equine ASCs were refractory to 5-azacytidine-induced upregulation of markers characteristic for the differentiation into heart cells. Hence, further studies are required to establish equine cardiomyocyte induction.

**Abstract:**

Physiological particularities of the equine heart justify the development of an in vitro model suitable for investigations of the species-specific equine cardiac electrophysiology. Adipose tissue-derived stromal/stem cells (ASCs) could be a promising starting point from which to develop such a cardiomyocyte (CM)-like cell model. Therefore, we compared abdominal, retrobulbar, and subcutaneous adipose tissue as sources for the isolation of ASCs applying two isolation methods: the collagenase digestion and direct explant culture. Abdominal adipose tissue was most suitable for the isolation of ASCs and both isolation methods resulted in comparable yields of CD45-/CD34-negative cells expressing the mesenchymal stem cell markers CD29, CD44, and CD90, as well as pluripotency markers, as determined by flow cytometry and real-time quantitative PCR. However, exposure of equine ASCs to 5-azacytidine (5-AZA), reportedly inducing CM differentiation from rats, rabbits, and human ASCs, was not successful in our study. More precisely, neither the early differentiation markers *GATA4* and *NKX2-5*, nor the late CM differentiation markers *TNNI3*, *MYH6,* and *MYH7* were upregulated in equine ASCs exposed to 10 µM 5-AZA for 48 h. Hence, further work focusing on the optimal conditions for CM differentiation of equine stem cells derived from adipose tissue, as well as possibly from other origins, are needed.

## 1. Introduction

Studies in equine cardiac electrophysiology have shown important electrophysiological differences for horses in comparison to other species, including humans. Thus, according to the pattern of propagation of electrical activity in the equine heart muscle, horses have been classified in the activation pathway group II, represented by pigs and including most animals [1]. This group has some important electrophysiological differences concerning the activation and propagation of electrical activity compared to humans (classified together with primates and carnivores in group I) [1]. Furthermore, the cardiac action potential lasts longer in horses than in other species and has an impressive ability to adapt to different heart rates [2]. These electrophysiological particularities distinguishing between horses and humans make it difficult to extrapolate results gained from one species directly to the other species. Therefore, species-specific studies are needed. Various in vitro models have been used to study cardiac electrophysiology in the past, including the two-electrode voltage clamp or patch clamp techniques with heterogeneously expressed channels in oocytes or mammalian cells [3,4,5,6]. Further myocardium wedge preparations have been used as in vitro models to study equine cardiac electrophysiology [3,4,5,6]. However, all these models have some limitations, including, for example, only one channel studied in the two-electrode voltage clamp or patch clamp technique. Technical difficulties with measurements hamper the wedge preparations. An equine cardiomyocyte (eCM)-like cell in vitro model would be extremely helpful to overcome some of these limitations.

Regenerative medicine and studies on the differentiation potential of pluri- or multipotent stem cells have made huge progress in the last few years [7,8,9]. However, most studies in equines have focused on orthopedic problems [10,11] and studies on other tissues are rare. Moreover, recent research identified adipose tissue as an abundant and easily accessible source of mesenchymal stem cells (MSCs) in comparison to bone marrow, umbilical cord or embryonic stem cells [12,13]. Furthermore, several protocols to isolate adipose tissue-derived stromal/stem cells (ASCs) have been described over the years in many species, including the horse [14,15,16,17,18,19,20,21,22,23,24]. Typically, ASCs, among other nucleated cells, were either isolated from the stromal vascular fraction (SVF) [15,18,19,20,21,22,23,25] or derived from a direct culture of explants of the adipose tissue [17,24,26,27].

Characteristics of MSCs have been demonstrated for ASCs [20,21,28,29], including multilineage differentiation potential (i.e., adipogenic, osteogenic, chondrogenic, myogenic, and neurogenic) in horses [18,19,20,21,22,23,30] and in other species [15,18,25,31]. Although earlier studies reported the potential of ASCs to differentiate between cardiomyocyte (CM)-like cells in rodents, rabbits, and humans [32,33,34,35], proof of CM differentiation of equine ASCs is lacking. A substantial number of these former studies used the DNA methylation inhibitor 5-azacytidine (5-AZA) to achieve the differentiation into CM-like cells [32,33,34,35]. The supposed mechanism behind the differentiation reported is the epigenetic activation or silencing of target genes [36]. Considerable variation in 5-AZA-mediated CM differentiation is reported, especially for human ASCs [32,35,37,38,39]. Nevertheless, results from these former studies [32,33,34,35] make ASCs a potential source for the development of an eCM-like cell model.

Adipose tissue is not uniform, and functional differences exist between white, visceral and brown adipose tissue [40,41]. It is well-known that the secretion of adipokines and pro-inflammatory mediators by the visceral adipose tissue contributes to the pathogenesis of human and equine metabolic syndrome [41,42]. Moreover, ASCs harvested from different sources of adipose tissue have recently been shown to present heterogeneous secretory profiles in culture and varying microenvironmental signals present at the original site might affect the phenotype of the ASCs [40,43]. Similarly, human and canine ASCs from different tissue sources displayed substantial differences in their differentiation and proliferation potential [32,44,45,46]. However, most studies in horses used subcutaneous (sc) adipose tissue as a starting material [18,19,20,21,22,23], and little is known about the differentiation potential of equine ASCs harvested from other sources of adipose tissue, such as abdominal (abd) adipose tissue [47,48].

Therefore, the current study aimed primarily to compare the yield and the morphological and molecular biological characteristics of equine ASCs derived from three different sources of adipose tissue applying two isolation methods. We hypothesized that the equine ASCs isolated by the two isolation methods of three sources of adipose tissue would show similar differences as those described for human and canine cells. The second aim was to investigate whether equine ASCs could be differentiated into eCM-like cells by using the DNA methylation inhibitor 5-AZA as a differentiation factor at a concentration used in other species.

## 2. Materials and Methods

### 2.1. Isolation and Cultivation of Equine ASCs

Regarding the harvesting of ASCs, abd, retrobulbar (rb), and sc adipose tissue was obtained from a total of 16 horses within 8 h post euthanasia at the Equine Hospital of the Freie Universität Berlin or after slaughter in commercial abattoirs. The reasons for euthanasia were unrelated to the present study. The horses were of different breeds, sexes, and ages (Supplementary Table S1). The abd adipose tissue was obtained from the properitoneal fat in the tela subserosa of the peritoneum near the linea alba; the rb adipose tissue was harvested after enucleation of the bulbus occuli; and the sc adipose tissue was obtained from the region next to the tail head over the superficial gluteal muscle. Sampling was performed in accordance with the German animal welfare legislation (reference StN 008/20–IV C 10).

After extraction, the adipose tissue samples (abd and sc: tissue specimen of approximately 9 g; rb: tissue specimen of approximately 6 g) were washed in sterile Dulbecco’s phosphate-buffered saline (DPBS) with calcium and magnesium (DPBS w Ca^2+^/Mg^2+^, Sigma Aldrich, Taufkirchen, Germany) and stored for 2 to 8 h at 4 °C in precooled DBPS w Ca^2+^/Mg^2+^, supplemented further with 1% penicillin-streptomycin (100 U/mL and 100 µg/mL, respectively, Sigma Aldrich, Taufkirchen, Germany) and 1% amphotericin B (2.5 µg/mL, BioWest, Riverside, CA, USA) in order to be transported to the lab of the Institute of Veterinary Physiology. In the lab, the adipose tissue was washed a second time, cut into smaller pieces (2 × 2 × 2 mm), and cleared of macroscopically visible blood vessels. Hereafter, ASCs were obtained either by collagenase digestion [18,19,20,21,22,23,25] or by the explant technique, described by others [17,24,26,27].

In brief, regarding the enzymatic digestion, the minced adipose tissue was incubated with 0.1% collagenase type I (1 mg/mL, Life Technologies GmbH, Karlsruhe, Germany) for 1 h at 37 °C under regular shaking and subsequently filtered through a 100-µm cell strainer (Geyer GmbH & Co. KG, Renningen, Germany). After a centrifugation step (260× *g*, 5 min, room temperature (RT), acceleration 2, brake 0), the cell pellet containing the SVF was seeded in 75 cm^2^ cell culture flasks (tissue culture flask 75, TPP Techno Plastic Products AG, Trasadingen, Switzerland) and incubated as indicated below in basal medium (B-M), composed of Dulbecco’s Modified Eagle Medium high glucose (DMEM high glc., Life Technologies GmbH, Karlsruhe, Germany) supplemented with 20% fetal bovine serum (FBS, Sigma Aldrich, Taufkirchen, Germany), 1% penicillin/streptomycin (100 U/mL and 100 µg/mL) and 1% amphotericin B (2.5 µg/mL). Typically, ASCs obtained from the SVF (ASCs-_SVF_) could be identified at approximately three to four days after seeding and were further cultivated. Utilizing the direct explant method, adipose tissue specimens were placed directly onto 12-well cell culture plates (tissue culture test plate 12, TPP Techno Plastic Products AG, Trasadingen, Switzerland) and incubated in 200 μl of B-M in order to allow the cell attachment to the bottom of the culture plates. A substantial number of explant-derived ASCs (ASCs-_EXP_) had attached and began to spread within one week, and any remaining adipose tissue was removed. Attached cells were propagated further under standard culture conditions at 37 °C, 5% CO_2_ and a humidified atmosphere with both methods. After the first passage, FBS content for both ASCs-_SVF_ and ASCs-_EXP_ was reduced from 20% to 10% (hereinafter the medium is referred to as expansion medium, E-M). The medium was changed every two to three days, and cells were passaged at 80–90% confluence. During passaging, the cells were detached by using trypsin-EDTA (Trypsin-EDTA solution, 0.25% Trypsin/0.02% EDTA, Sigma Aldrich, Taufkirchen, Germany), according to the procedure described in Jurek et al. [49].

At this point, the success of harvesting precursor cells from the different adipose tissues by both isolation methods was analyzed by using a binary isolation success scoring system (0 points: nongrowth of cells, isolation not possible; 1 point: growth of cells, successful isolation; contamination was not taken into account). Each isolation was scored, and points were awarded for the isolation technique and adipose tissue localization in cells of passage 0 (P0). The 12 wells used for isolation were summed up in an overall score for the explant technique, whereas each isolation in the enzymatic digestion was done in a single-cell culture flask and scored.

In order to have sufficient cells available for all further experiments, the cells were expanded up to passage 2 or 3 (P2–P3), trypsinized, counted, and cryopreserved in E-M supplemented with 5% DMSO (2 × 10^6^ cells/mL, dimethyl sulfoxide, sterile filtered, Sigma Aldrich, Taufkirchen, Germany). The expanded ASCs were further characterized morphologically by transmitted light microscopy, immunophenotypically by flow cytometry, and molecular biologically by real-time quantitative PCR.

### 2.2. Antibody Staining and Flow Cytometry

In accordance with the criteria defined by the International Society of Cellular Therapy (ISCT) for work with human MSCs [28], the purity of the equine ASCs-_EXP_ and ASCs-_SVF_ populations was surveyed based on a stem cell-specific profile of the surface marker expression adapted for horses [21,29,50,51]. The ASCs-_EXP_ or ASCs-_SVF_ derived from the most suitable adipose tissue localization were detached from cell culture flasks by Accutase Cell Detachment Solution (PAN Biotech GmbH, Aidenbach, Germany), washed in FACS buffer (DPBS, Pan Biotech GmbH, Aidenbach, Germany, supplemented with 0.2% bovine serum albumin (BSA) and 2 mM EDTA) and counted with a Neubauer chamber. Cells were transferred at a concentration of 100,000 cells per well to V-shape 96-well plates (Greiner Bio-one, Kremsmünster, Austria) and stained in phosphate-buffered saline (PBS) with 0.2% BSA for 10 min on ice with the following monoclonal antibodies: CD45 (clone F10-89-4, biotin; BIO-Rad Laboratories GmbH, Feldkirchen, Germany), CD34 (clone 581, PE phycoerythrin; BioLegend, San Diego, CA, USA), CD29 (clone TS2/16, APC allophycocyanin; BioLegend, San Diego, CA, USA), CD44 (clone IM7, PerCP peridinin-chlorophyll protein; BioLegend, San Diego, CA, USA), CD105 (clone SN6, PE-Cy7 phycoerythrin-cyanine7; ThermoFisher Scientific Inc., Waltham, MA, USA), and CD90.1 (clone OX-7, Pacific Blue; BioLegend, San Diego, CA, USA). Streptavidin-Alexa Fluor 488 (BioLegend, San Diego, CA, USA) was used as a secondary conjugate and dead cells were excluded by using the fixable viability dye eFluor780 (ThermoFisher Scientific Inc., Waltham, MA, USA). The following isotype controls were used to verify the specificity of antibody binding: clone OKT3 (biotin; ThermoFisher Scientific Inc., Waltham, MA, USA), MOPC-173 (PE; BioLegend, San Diego, CA, USA), and P3.6.2.8.1 (APC and PE-Cy7; ThermoFisher Scientific Inc., Waltham, MA, USA). Similar to other studies (e.g., Paebst et al. [29]), equine peripheral blood mononuclear cells (PBMC) were included in the flow cytometry approaches as additional controls in order to prove the binding of the antibody clones applied. To that end, EDTA blood was harvested from five donor horses through venipuncture at the jugular vein and kept on ice until PBMC isolation

The samples were diluted 1:2 in 0.9% NaCl and transferred into tubes filled with 12.5 mL human Pancoll (PAN-Biotech GmbH, Aidenbach, Germany). Following centrifugation (800× *g*, 20 min, RT, acceleration 1, brake 0), the PBMC were collected from the interphase. After a further washing step, the pellets were suspended in FACS buffer, counted in a CASY Cell Counter (Roche-Innovatis, Reutlingen, Germany) and subjected to single and combined stains with the anti-target antibodies or respective isotype controls, as specified above.

Cells were acquired on a BD FACSCanto II (BD Biosciences, NJ, USA) and analyzed by using FlowJo software (version 9.9.6, Tree star Inc., Ashland, OR, USA).

### 2.3. Cardiomyogenic Induction

**Figure 1:**
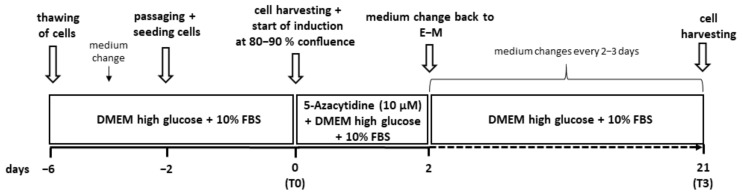
Timeline showing the protocol for the induction experiment with 5-azacytidine (5-AZA). E-M, expansion medium; FBS, fetal bovine serum; DMEM, Dulbecco’s Modified Eagle Medium.

Based on the results of the flow cytometry and the analysis of isolation success, the abd ASCs-_SVF_ were used for this part of the study. When abd ASCs-_SVF_ reached 80–90% confluence (time point T0, before induction), the cells were incubated with E-M containing 10 µM 5-AZA for 48 h (Figure 1). Thereafter, the cells were further cultivated in E-M with medium changes every two to three days and harvested on day 21 (time point T3, three weeks after induction).

At this time point (T3), the expression of pluripotency and cardiac markers was investigated by SYBR Green real-time quantitative PCR and compared to the expression in non-induced abd ASCs-_SVF_ at day 0 (T0). Untreated control cells cultivated in E-M until T3 served as negative controls (nc). Cells were cultivated in uncoated 75 cm^2^ cell culture flasks (tissue culture flask 75, TPP Techno Plastic Products AG, Trasadingen, Switzerland). Cell morphology (CM appearance, spontaneous beating of cells) was subjectively assessed every two to three days by light microscopy within the induction period of three weeks. Each experiment was conducted with abd ASCs-_SVF_ of five horses in two experimental runs to ensure reproducibility.

### 2.4. Real-Time Quantitative PCR

Real-time quantitative PCR was performed at T0 and T3 in order to analyze and compare the expression of pluripotency markers *OCT4*/*POU5F1* and *MYC*, cardiac markers *GATA4*, *NKX2-5*, *TNNI3*, *MYH6*, and *MYH7*, and the muscle marker *MYF6* (for excluding myogenic differentiation) [52,53,54]. The successful cardiomyogenic induction would be expected to be accompanied initially by the downregulation of the pluripotency markers typical for MSCs (*OCT4*/*POU5F1*, *MYC*) [38], and the upregulation of the early cardiogenic transcription factors *GATA4* and *NKX2-5* among others [54,55]. Subsequently, structure proteins, such as the alpha isoform (gene *MYH6*) or the beta isoform of the myosin heavy chain (gene *MYH7*), and cardiac troponin (gene *TNNI3*), would be expressed [54,56]. Regarding the real-time quantitative PCR, the RNA extraction of T0 and T3 cells was performed by using the extraction kit Nucleospin RNA II (Macherey Nagel GmbH & Co. KG, Düren, Germany), according to the manufacturer’s instructions. The quantity of RNA extracted and the degree of impurities caused by salts and proteins were analyzed spectrophotometrically with a Nanophotometer™ (Implen, GmbH, Munich, Germany). The quality of the RNA was assessed by the quotient of ribosomal RNA 18S/28S microelectrophoretically via Agilent-Bioanalyzer-Nano (Agilent RNA 6000 Nano Kit, Agilent Technologies, Böblingen, Germany) as a “lab-on-a-chip” method. The cDNA synthesis was performed with the iScript™ cDNA Synthesis Kit (BIO-Rad Laboratories GmbH, Feldkirchen, Germany), following the manufacturer’s instructions. Primers, listed in Table 1, were designed based on the gene database NCBI (Primer-BLAST) and software Primer3Plus.

After reverse transcription, specific cDNA could be amplified by means of the forward and reverse primers by using conventional PCR, which was subsequently sequenced (Microsynth Seqlab GmbH, Göttingen, Germany). The SYBR Green real-time quantitative PCR was carried out by using an iCycler iQ™ (BIO-Rad Laboratories GmbH, Feldkirchen, Germany), according to the following conditions: 1 cycle at 95 °C for 2 min (denaturation) and 40 cycles in three steps at 95 °C for 5 s, at 55 °C for 10 s and at 72 °C for 20 s (amplification). Because SYBR-Green can lead to unspecific products unless probe-based real-time quantitative PCR is used, a melting curve including 80 cycles from 55 °C in 0.5 °C in increments of 10 s each per temperature step was appended after each 40-cycle PCR run. The DNA contaminations were excluded based on nc containing water (H_2_O) and samples without reverse transcriptase (NRT). A composite sample taken from all samples (inter-run-control) with a reference gene (*GAPDH*) on each plate was detected and, after passing all plates, was normalized thereon to avoid plate differences of various PCR runs. A positive control sample was entrained on each PCR plate, depending on the gene.

Investigations included mRNA quantification in atrial and ventricular tissue for cardiac-specific genes, in muscle tissue for muscle markers, and in ASCs for pluripotency markers as reference populations. Respective samples were recovered from heart muscle tissue obtained directly post-mortem from six euthanized horses provided by the Department of Veterinary Clinical Sciences Section of Large Animal Medicine and Surgery of the University of Copenhagen and muscle tissue harvested directly post-mortem from two donor horses provided by the Equine Clinic of Freie Universität Berlin. Relative quantification using the 2^−ΔΔCt^ method according to Livak and Schmittgen [57,58] revealed that cardiac markers and the muscle marker were amplified with high Ct values and were, therefore, in the negative area of gene regulation. Hence, the Livak method was not used.

### 2.5. Statistical Analyses

Data analysis was performed visually and with commercially available statistical software (Graph Pad Prism, Version 9.1.2 for Mac, GraphPad Software, San Diego, CA, USA and IBM SPSS, Version 27, Armonk, NY, USA). The isolation success of ASCs was assessed with linear mixed models and defined as the target. The source of adipose tissue, isolation methods and the interaction between the source of adipose tissue and the isolation method were fixed factors and horse was the random factor. Statistical tests were performed for the analysis of the results of the real-time quantitative PCR only when Ct mean values of individual markers and treatments were more than four cycles away from the mean values of the nc, NRT and H_2_O. In these cases, a one-way ANOVA with Tukey’s HSD or Hochberg post-hoc test for multiple comparisons was applied. Results are shown as means ± standard deviation. Statistical significance was set at *p* < 0.05.

## 3. Results

### 3.1. Isolation and Cultivation of Equine ASCs

**Figure 2:**
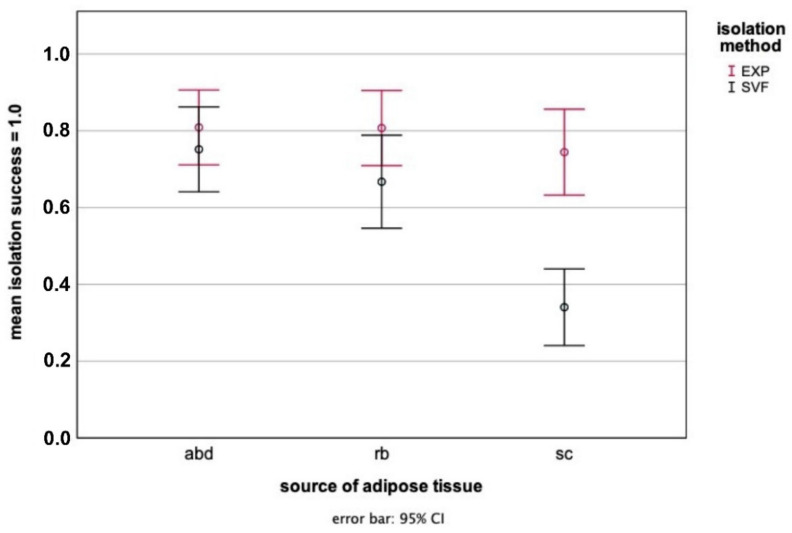
Isolation success of adipose tissue-derived stem/stromal cells (ASCs) harvested by explant culture (ASCs-_EXP_, red) and collagenase digestion (ASCs-_SVF_, black) from abdominal (abd), retrobulbar (rb), and subcutaneous (sc) adipose tissue based on the isolation success scoring.

Self-renewable ASCs could be obtained from abd, rb, and sc adipose tissue with both the explant culture and collagenase digestion methods. Cells were plastic-adherent with typical fibroblast-like morphology. Although ASCs-_SVF_ attached mostly three to four days after seeding to the bottom of cell culture flasks, ASCs-_EXP_ grew out of the explants within one week. The linear mixed model used to compare the isolation success of ASCs showed a significant influence of the source of adipose tissue (*p* = 0.003) but not of the isolation method (*p* = 0.12). Furthermore, there was no significant interaction between the source of adipose tissue and the isolation method (*p* = 0.19). The isolation of abd ASCs-_EXP_ and rb ASCs-_EXP_ was comparably successful, but the abd adipose tissue appeared to be most suitable for the isolation of ASCs-_SVF_, as shown in Figure 2. Furthermore, ASCs harvested from sc adipose tissue by both isolation procedures displayed the lowest isolation success, i.e., fewer cells adhered (Figure 2). In the following, the characterization of the ASCs and the induction experiments were conducted only with ASCs obtained from abd adipose tissue, as this adipose tissue localization showed the best isolation success overall. Furthermore, in order to facilitate comparison with other studies, cells obtained from the SVF were used for the induction experiments because the isolation method did not seem to influence the isolation success.

### 3.2. Flow Cytometry

**Figure 3:**
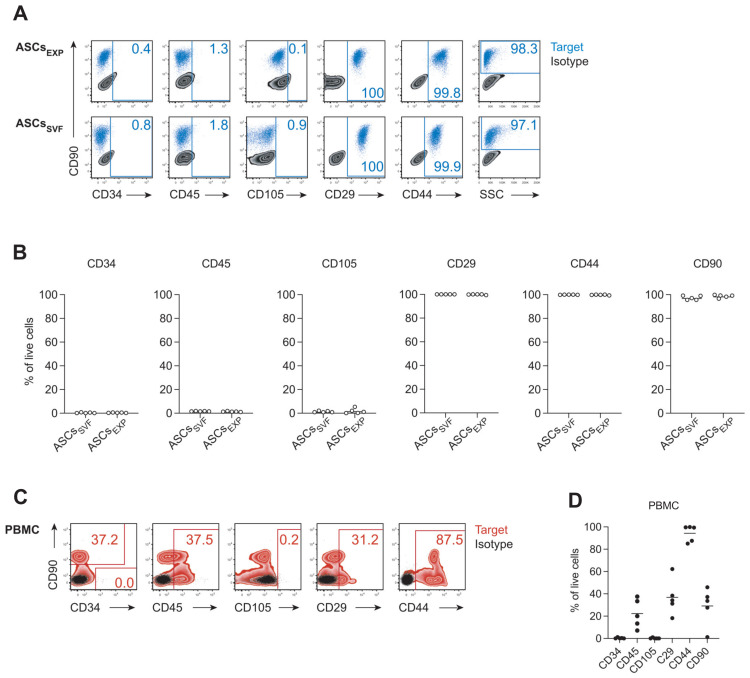
Characterization of equine abdominal adipose tissue-derived stem/stromal cells (ASCs) by flow cytometry. (**A**) Surface marker expression by ASCs-_EXP_ and ASCs-_SVF_. Representative overlay plots including fully stained cells (target, blue) and isotype controls (black) are shown. (**B**) Marker expression by ASCs generated with the two protocols. Data pooled from two (ASCs-_SVF_) and four (ASCs-_EXP_) independent experiments, each performed with cells from one to three individual donors. (**C**) Equine peripheral blood mononuclear cells (PBMC, *n* = 5) were used as positive controls to survey the binding of the antibodies applied for the characterization of equine stem cells. Representative overlay plots report fluorescence signals detected with fully stained cells (target, red) and isotype controls (black). The CD90 was stained in all samples. Numbers indicate the percentage of cells expressing the target molecules. (**D**) Marker expression by PBMC isolated from five donors. Gating strategies are reported in Supplementary Figure S1. FSC, forward scatter; SSC, side scatter.

Flow cytometry indicated similar viability for abd ASCs-_EXP_ (mean ± SD, 70.4 ± 10.8%) and abd ASCs-_SVF_ (69.3 ± 7.75%) and confirmed the absence of the negative markers CD34 (abd ASCs-_EXP_: 0.44 ± 0.46%; abd ASCs-_SVF_: 0.38 ± 0.30%) and CD45 (abd ASCs-_EXP_: 1.42 ± 0.41%; abd ASCs-_SVF_: 1.40 ± 0.64%) on the surface of abd ASCs-_EXP_ and abd ASCs-_SVF_. Furthermore, both cell preparations uniformly expressed the typical stem cell surface marker molecules CD29 (abd ASCs-_EXP_: 99.7 ± 0.42%; abd ASCs-_SVF_: 100 ± 0.00%), CD44 (abd ASCs-_EXP_: 99.9 ± 0.26%; abd ASCs-_SVF_: 99.9 ± 0.05%) and CD90 (abd ASCs-_EXP_: 96.7 ± 4.20%; abd ASCs-_SVF_: 96.4 ± 3.23%) (Figure 3A,B). The surface marker CD105 reported to be expressed at variable levels by equine stem cells in earlier studies [20,21,23,29,47,50,51,59] was not detectable in the present study (abd ASCs-_EXP_: 1.68 ± 2.01%; abd ASCs-_SVF_: 1.18 ± 0.77%) (Figure 3A,B). Equine PBMC stained in parallel to ASCs confirmed the binding patterns expected for CD90 (expressed by peripheral T cells; PBMC: 29.1 ± 17.0), CD45 (expressed in several splice variants by all hematopoietic cells; PBMC: 22.3 ± 12.9%), CD29 (b1 integrin expressed to varying degrees on hematopoietic cells; PBMC: 36.8 ± 16.1%), and CD44 (upregulated on activated B and T cells; PBMC: 94.3 ± 7.4%). By contrast, CD34 (primarily expressed by hematopoietic stem/progenitor cells) and CD105 (expressed by endothelial cells and activated macrophages, reported earlier to be present on equine stem cells) were largely absent from equine PBMC (Figure 3C,D). Specific background signals of primary antibodies could be excluded by using isotype controls.

### 3.3. Cardiomyogenic Differentiation

#### 3.3.1. Cell Morphology

**Figure 4:**
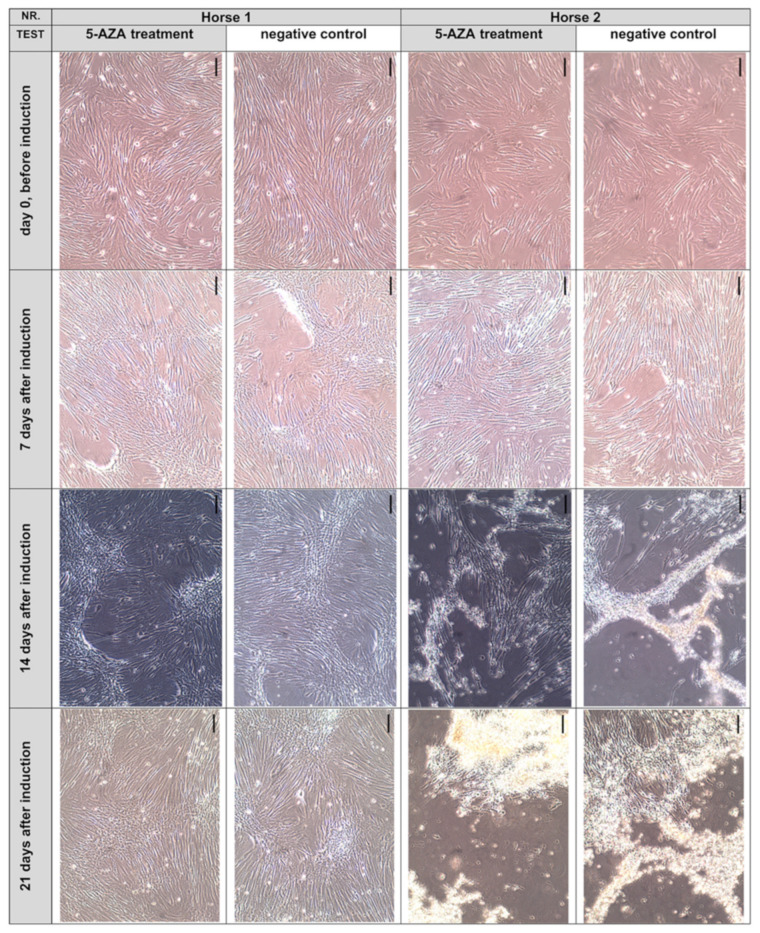
Morphology of abdominal adipose tissue-derived stem/stromal cells (ASCs) obtained by collagenase digestion before (day 0) and after (day 7, 14 and 21) exposure to 5-azacytidine (5-AZA) (exemplary for *n* = 2 donors). The cells of horse 1 exhibited the typical fibroblast-like morphology and moderate “dome” formation. Cell detachment was seen in horse 2 after two to three weeks as well as atypical small cells indicative of this. No differences between the induced and negative control cells were visible in either horse. Scale bar: 0.1 mm.

Previous studies [33,34,35,38] reported morphological changes of ASCs after exposure to 5-AZA, including the appearance of a stick-like morphology or ball-like cells, the formation of cell-to-cell connections, and myotube-like structures. However, such morphological modifications were absent in our cultures of equine abd ASCs-_SVF_ derived from passage 3 or 4 and exposed to 5-AZA for 48 h (Figure 4). Furthermore, no spontaneous beating activity was seen.

The abd ASCs-_SVF_ of two out of five donors maintained their spindle-shaped, fibroblast-like cell morphology, regardless of whether they were exposed to 5-AZA or not. A moderate, incipient “dome” formation, indicative of overconfluent growth, was detected in cultures derived from two out of five donors, and a dome formation in cells of the other three out of five donors was partly more severe. These cells cultivated in uncoated culture flasks showed a partially strong cell detachment and cell death independent of the treatment. We identified atypical small cells with a bright shining nucleus and atypical mitotic-like cells by light microscopy as indicators of cell detachment. Subjectively, no differences in the morphological appearance between the exposed and nonexposed abd ASCs-_SVF_ could be detected at any time. The following Figure 4 illustrates a survey of transmitted light microcopy images.

#### 3.3.2. Reverse-Transcription Real-Time Quantitative PCR

The RNA extracted from all samples showed a high quality and quantity, as proven via spectralphotometric measurements in a Nanophotometer™ (Ratio: close to 2) and investigations in a Bioanalyzer-Nano (RIN value: close to 10; desired value: 6–10).

Gene expression of the two pluripotency markers *OCT4*/*POU5F1* and *MYC* was observed at T0 and, together with the morphological and immunophenotypical results, confirmed that the cells harvested were ASCs. At T3, both markers were significantly downregulated, both in 5-AZA-induced cells (*OCT4*/*POU5F1*: T0 vs. T3_nc: *p* < 0.001; *MYC*: T0 vs. T3_nc: *p* = 0.011) and non-induced controls (*OCT4*/*POU5F1*: T0 vs. T3_ind: *p* = 0.001; *MYC*: T0 vs. T3_ind: *p* < 0.001).

Statistical tests were applied when the mean Ct values in the real-time quantitative PCR of a given marker or treatment differed from the mean values of negative controls (nc), NRT and H_2_O by at least four cycles, as described above.

Contrary to expectations, the 5-AZA treatment did not result in an upregulation of cardiac marker genes *GATA4*, *NKX2-5*, *TNNI3*, *MYH6*, and *MYH7* compared to positive controls (atrial and ventricular heart muscle tissue, respectively) and, therefore, the cardiomyogenic induction was unsuccessful in our setting. Statistical tests were not performed in this part of the experiments because the Ct mean values of individual markers and treatments were consistently less than four cycles away from the mean value of the nc, NRT and H_2_O. A differentiation towards a myogenic cell line could be excluded as no upregulation of the muscle marker gene *MYF6* was observed.

**Figure 5:**
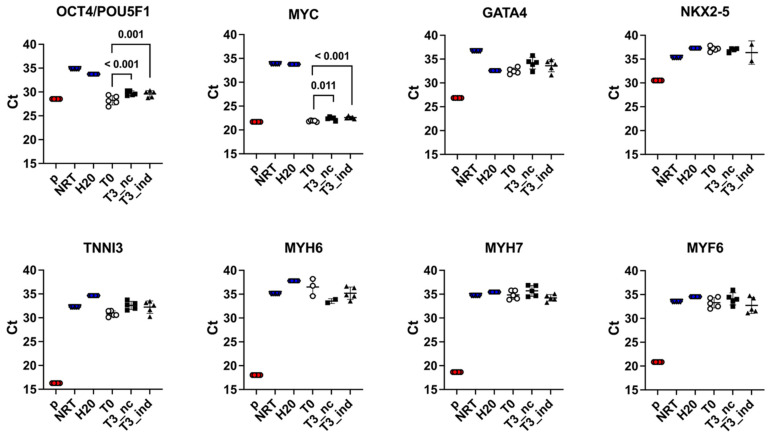
Results of the SYBR Green real-time quantitative PCR for the pluripotency, cardiac, and myogenic markers expressed in equine adipose-derived stem/stromal cells (ASCs) after exposure to 5-azacytidine (5-AZA) for 48 h. Gene expression at T0 (nonexposed) and T3 (exposed T3_ind, induced and T3_nc, negative control) as well as positive (*p*, red color symbols) and negative control (nc as NRT and H_2_O controls, blue color symbols) are shown. Dot plots represent Ct mean values with standard deviation from two experimental runs (*n* = 5 donors). One-way ANOVA (post hoc tests: Hochberg, Tukey’s HSD) was used for the statistical analysis. Statistical significance was set at *p* < 0.05.

The Ct mean values of the real-time quantitative PCR with standard deviation are shown in Figure 5 as scatter dot plots and described in detail in the Supplements (Supplementary Table S2).

## 4. Discussion

The main finding of our study was that ASCs could be isolated from all sources of adipose tissue sampled in horses, as expected [18,19,20,21,22,23,24,47]. In line with results from human and canine studies [32,44,45,46], we could show that the source of adipose tissue influences the isolation success. The ASCs-_SVF_ from abd adipose tissue showed a better isolation success than the ASCs-_SVF_ harvested from the two other sources in our study (i.e., retrobulbar and subcutaneous adipose tissue). On the other hand, the isolation success for both isolation method was equivalent in our experiments. Furthermore, the cell viability determined by flow cytometry for ASCs-_SVF_ and ASCs-_EXP_ was similar, confirming that both methods can be used for the isolation of ASCs in horses [24], as described for other species [17,24,26,27]. However, in contrast to studies with rodent or rabbit cells [33,34,60], we were not successful in differentiating ASCs-_SVF_ into eCM-like cells after exposure to 5-AZA for 48 h.

The first aim of the present study was to compare different sources of adipose tissue and different isolation methods for the isolation of equine ASCs. Adipose tissue is abundant and easily accessible compared to bone marrow and umbilical cord cells [12,13]. This accessibility confers a great potential on to ASCs to establish in vitro cell models, whereas further criteria, such as the immunogenicity of the cells used, have to be considered for an in vivo allogeneic application to avoid immunological rejection [43].

Some former studies in humans and horses [18,19,20,21,22,23,31] have assessed the similarity of ASCs to other MSCs, their differentiation potential and their phenotypical characteristics [12,13]. Concerning the source of adipose tissue, the sc location for harvesting adipose tissue to isolate ASCs has been widely used in horses [18,19,20,21,22,23,30], whereas the abd source has been used more often in cats [25,61], dogs [45,46,62], and humans [32]. Regarding horses, two studies reported the use of adipose tissue harvested abdominally around the mesentery of the small colon [48], in the linea alba and in lipomas [47]. Nevertheless, direct comparisons are sparse [41,42]. The structural and functional heterogeneity of adipose tissue has been shown in vivo and in vitro [13,40,41,44]. Considering the higher isolation success from the abd location, our results are in line with studies showing that the number of cells isolated from abd adipose tissue was higher than in those harvested at the tail head and that those cells had better viability [44,45,47,48]. However, these results should be confirmed by including a larger number of samples and a more homogenous population of horses with regard to age and breed. Special care should be taken regarding the breed and body conditions of the donor horses. It is well-known that easy keeper type horses have a particular tendency to adiposity and insulin dysregulation [63,64]. Such factors might influence the differentiation potential of MSCs, as has been suggested recently [65]. The difference between the rb and sc adipose tissue is more difficult to explain, but could be related to the fact that mainly structural white adipose tissue can be found at these locations. White adipose tissue has less proliferation potential compared to adipose tissue harvested from the abdomen or from a peri- or epicardial origin based on exposure to a completely different microenvironment [32,44].

Regarding the isolation methods, the collagenase digestion is an established method of isolating ASCs and has been used in different species including horses [15,18,19,20,21,22,23,25]. The explant method has been described more recently as a simple method by which to isolate human [26,27,66], equine [24], and bovine MSCs [17]. In a direct comparison, the fibroblast-like aspect, the adhesion to plastic and the isolation success were similar in cells obtained with both methods in the present study, which is in accordance with results of a previous study in horses [24]. Furthermore, the expression of CD markers was similar in both populations of cells in our experiments. When comparing the CD profile of equine cells, some differences have to be considered. According to the criteria defined by the International Society of Cellular Therapy (ISCT), ≥95% of human MSCs express the surface markers CD105, CD73, and CD90, and MSCs largely lack the expression of CD45, CD34, CD14, CD11b, CD79α, CD19, or HLA-DR (present in ≤2% of the cell population) [28]. Regarding equine MSCs, previous work defined the expression of CD29, CD44, and CD90 and the absence of CD34 and CD45 as a typical marker profile [20,21,23,29,47,59]. In compliance with these earlier works on equine MSCs, both ASCs-_EXP_ and ASCs-_SVF_ generated in the present study displayed MSCs characteristics evident in the homogenous expression of CD29, CD44, and CD90 and the lack of CD34 and CD45. On the other hand, a difference to human MSC concerns CD 105. Although several earlier studies reported the varying expression of CD105 by equine MSCs [20,21,23,29,47,50,51,59], our study confirms the work by Ranera et al. [21] reporting the lack of CD105 surface expression on bone marrow MSCs and ASCs. These data, as well as the cell viability of about 70% determined for both abd ASCs-_EXP_ and abd ASCs-_SVF,_ confirm the explant method as equivalent to the digestion method for isolating ASCs from horses [24]. Moreover, our results are in line with results from a study comparing the two methods in respect to ASCs generation from human synovial tissue [27]. In summary, ASCs-_EXP_ and ASCs-_SVF_ were highly comparable concerning expression profiles of MSC markers and, different from previous studies [12,13,24], cells obtained by the SVF technique did not comprise elevated numbers of hematopoietic lineage cells, endothelial cells, pericytes, or other stromal cells in our hands [12,13,24].

These results were obtained with a time frame that extended up to 8 h for a few samples; however, even these cells still seemed to have good viability. Good viability and proliferation was shown previously for bone marrow MSCs harvested 30 min after euthanasia in horses [51]. Taken together, this could indicate that viable MSCs might be harvested much longer after death than expected. However, this topic should be investigated in studies specifically designed to address this question.

In addition to the flow cytometry approach, we also monitored the expression of a group of well-known transcription factors: *OCT4*/*POU5F1*, *NANOG*, *SOX2*, and *MYC*, which were shown to be associated with pluripotency and self-renewal of especially embryonic stem cells [59,67,68,69,70]. Confirming earlier reports of *OCT4*/*POU5F1* expression in equine ASCs [69,71] and *MYC* expression in human ASCs [72], we detected the expression of both transcription factors in ASCs-_SVF_ at T0. These two pluripotency markers showed downregulation over three weeks in induced and non-induced cells. Similar downregulation of pluripotency markers have been reported by Safwani et al. [38]. Although they reported no change in the expression of *OCT4*/*POU5F1*, they showed a downregulation over time of *SOX2* and *NANOG* when cells were exposed to 5-AZA [38]. The reduction of the stem cell markers in both the stimulated and non-stimulated cells, as seen in our experiment, could be a sign of senescence of cells and, therefore, indicate a loss of viability, although good viability has been reported until passage 4 or 10 for canine ASCs [45,46,62] or passage 7 for human ASCs [73].

In the second part of the study, we investigated the reaction of equine ASCs to the incubation with 5-AZA. Several previous studies reported the 5-AZA-driven differentiation of ASCs into CM-like cells in cells isolated from rats, rabbits, and, inconsistently, from human ASCs. The differentiation was documented by showing morphological changes [33,34,35,37] and the upregulation of heart-specific genes, such as myosin heavy chains, sarcomeric alpha-actinin, connexin 43, or heart-specific ion channels [33,34,35,37,60]. In one report, even the spontaneous beating of cells was described [34]. To see whether abd ASCs-_SVF_ generated in our study responded in a similar manner, we exposed the cells to 10 µM 5-AZA for 48 h. Under these conditions, we did not observe any upregulation of cardiac differentiation markers, morphological changes or beating behavior. It is notable that conflicting reports exist on the effects of 5-AZA on ASCs isolated from human tissue. One study showed that ASCs exposed to 6 μM 5-AZA for 24 h responded with an upregulation of heart-specific genes [37], and exposure for 48 h was required according to another study [35]. By clear contrast, other studies applied 5 to 10 μM 5-AZA for 24 h (or even repeatedly) and found no evidence of any upregulated expression of cardiac genes [32,38,39]. Although the reason for these conflicting results is not entirely clear, it has to be borne in mind that 5-AZA is an unspecific differentiation agent [36]. As an unspecific gene activator, 5-AZA has been used in combination with resveratrol to avoid the aging of ASCs [74], and this result might show a promising new application for 5-AZA in equine regenerative medicine [75]. However, the unspecific activation of gene expression could be an explanation for the variable success that has been reported with 5-AZA in human cells and in our equine cells; more cardiac-specific transformation protocols should probably be investigated in the future [12,56,76,77]. As several reports indicated impaired cell proliferation and elevated cell death for prolonged or repeated exposure of ASCs to 5-AZA as well as in response to a higher concentration [34,39], we did not expose our cells to a higher concentration or beyond 48 h. In line with the unaltered morphology and the lack of cardiac differentiation markers, we found no evidence of the elevated expression of *GATA4* and *NKX2-5*, two transcription factors known to be essential in early stages of cardiac development [54,55,68]. This result is also in line with other studies reporting little or no impact of 5-AZA on the differentiation potential of human ASCs into CM-like cells. Furthermore, constitutively higher expression of *GATA4* was shown in epicardial ASCs, possibly indicating a slightly better differentiation potential in these cells compared to ASCs isolated from omental adipose tissue [32]. Differences in the differentiation potential were also reported in cells isolated from dogs, in which the number of MSCs isolated was higher in omental adipose tissue [45], but the pluripotent differentiation potential was maintained longer in subcutaneously harvested ASCs [46]. These different results highlight that differentiation depends partially on the origin of the adipose tissue and probably also on the microenvironment of the cells harvested. In the absence of an upregulation of early cardiac genes, we could not see an upregulation of the more specific cardiac genes (*TNNI3*, *MYH6*, and *MYH7*) in contrast to former studies [33,34,35,37,60].

Apart from the origin of adipose tissue, species, individual characteristics, or culture technics might have hampered our results and should be discussed as limitations. Concerning species, studies in rodents, where it is much easier to harvest tissue from a homogenous laboratory-bred population, have shown much better results, as there are several studies where both the early and specific cardiac markers were upregulated in experiments using the same or other induction protocols, independently of the isolation technique [33,34,56,60]. Secondly, it has recently been highlighted that the characteristics of MSCs depend on the physiological status of the donors [65]. The MSCs have shown signs of early senescence in the case of cells harvested from subjects with diabetes or metabolic syndrome [65]. Therefore, the inhomogeneous population from which our ASCs have been harvested must be considered as a limitation that should be optimized in further studies. Third, the culture conditions seem to be a non-negligible factor. The different culture mediums used in the mentioned studies could explain parts of the discrepancies in the reported results [32,35,37,38,39,78,79] and further an improvement of the cardiomyogenic differentiation potential could be obtained by using laminin-coated culture dishes, as shown for human ASCs [80]. In addition, an increasing passage number might reduce the proliferation and differentiation potential. The passage number in our studies was similar to that applied in other successful studies [32,33,35,37,60]. However, it is still possible that the high passage number (>passage 6) with possibly reduced proliferation or differentiation potential, as reported in men and dogs [45,46,62,73], might have contributed to the reduced transdifferentiation success in the present and previous studies [38]. Lastly, the cardiomyogenic differentiation potential might vary depending also on the origin of MSCs. Although in some species bone marrow-derived MSCs and ASCs could be successfully differentiated into CM-like cells after exposure to 5-AZA, their umbilical cord counterparts did not always show such potential [33,34,35,37,78,79,81].

## 5. Conclusions

Adipose tissue-derived stem cells could be isolated from different sources of adipose tissue in horses, as expected based on previous studies [18,19,20,21,22,23,24,32,44,45,46,47]. In line with these studies from human and dogs [32,44,45], the ASCs isolated from abd adipose tissue showed a better isolation success than ASCs harvested from the two other sources. The cells isolated showed immunological and molecular biological characteristics of MSCs; however, they could not be transformed into eCM-like cells with our induction protocol that was based on protocols for successful CM differentiation in other species [33,34,35,37,60]. Therefore, further studies will be needed to optimize sources of ASCs, protocols and laboratory methods to achieve the successful induction of eCM differentiation.

## Figures and Tables

**Table 1:** List of primers used for SYBR Green quantitative reverse-transcription real-time PCR.

Gene	mRNA	Gene ID	Sequence 5′-3′	Exon No	Intron Length	Product Length
***ACTB* ^1^**	NM_001081838.1	100033878	For: GCCAACCGCGAGAAGATGAC	2	448	124
			Rev: AGTCCATCACGATGCCAGTG	3		
***GAPDH* ^1^**	NM_001163856.1	100033897	For: AAGAAGGTGGTGAAGCAGG	9	86	116
			Rev: GCATCGAAGGTGGAAGAGTGGG	10		
***RN18S* ^1^**	NW_019643269.1	100861557	For: ACTCACACGGGAAACCTCAC	1	0	122
			Rev: AACCAGACAAATCGCTCCAC	1		
***MYC* ^2^**	XM_001497991.1	100068097	For: CAGCGACTCTGAAGAAGAAC	1	1069	241
			Rev: ACTGTCCAACTTAGCCCTC	2		
***OCT4*/** ***POU5F1* ^2^**	XM_001490108	100050785	For: AGCAATTTGCCAAGCTCC	2	633	235
			Rev: GTCTCTGCTTTGCATATCTCC	3–4		
***GATA4* ^3^**	XM_023636259.1	100065126	For: CAGAAAACGGAAGCCAAAGAAC	4	2747	218
			Rev: ACATCGCACTGACCGAGAAC	6		
***NKX2-5* ^3^**	XM_005614765.3	100069632	For: AAGGACCCTCGAGGCGATAA	1	1508	247
			Rev: ACCAGATCTTGACCTGCGTG	2		
***TNNI3* ^3^**	NM_001081904.1	100034065	For: TGGATGAGGAGAGATACGATG	6	547	101
			Rev: CTTAAACTTGCCCCGAAGG	7		
***MYH6* ^3^**	XM_023622391.1	111767446	For: GCGCATCGAGTTCAAGAAG	18	1254	188
			Rev: TGATACGCCCAAACTCCTCC	19		
***MYH7* ^3^**	NM_001081758	791234	For: TGAGAAGGGCAAAGGCAAG	15	385	129
			Rev: ATGATGCAACGCACGAAG	16		
***MYF6* ^4^**	NM_001317257.1	100050603	For: CAGCTACAGACCCAAGCAAGA	1	539	202
			Rev: AGGAGAGTTTGCGTTCCTCC	3		

^1^ Reference gene, ^2^ stem cell gene, ^3^ heart muscle gene, ^4^ muscle gene.

## Data Availability

All data is contained within the manuscript and its supplementary material.

## Supplementary Materials

The following supporting information can be downloaded at: https://www.mdpi.com/article/10.3390/ani12162049/s1, Table S1: Study collective; Table S2: Numeric results of the real time quantitative PCR before and after exposition to 5-azacytidine; Figure S1: Gating strategies applied for the characterization of equine adipose tissue-derived mesenchymal stem cells (ASCs) by flow cytometry.
