# Supplementary material for: Comparison of Sources and Methods for the Isolation of Equine Adipose Tissue-Derived Stromal/Stem Cells and Preliminary Results on Their Reaction to Incubation with 5-Azacytidine
Source: Animals (Basel). 2022 Aug 11;12(16):2049. doi: 10.3390/ani12162049 (PMC9404420; doi:10.3390/ani12162049)

Supplementary Figure S1 Gating strategies applied for the characterization of equine adipose tissue-derived mesenchymal stem cells (ASCs) by flow cytometry. EXP, explant method; FSC, forward scatter; PBMC, peripheral blood mononuclear cells; SSC, side scatter.

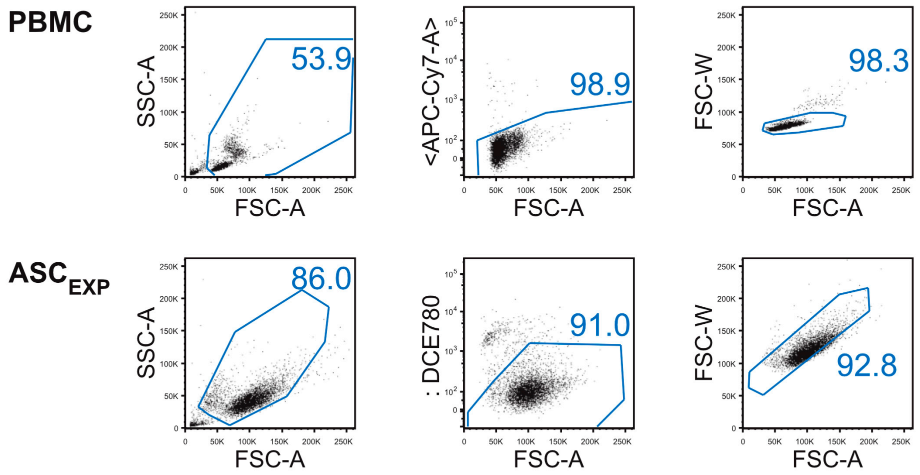

Supplement: Supplementary file 1 [file animals-12-02049-s001.zip › Supplementary Figure S_5AZA equine ASC_Gating strategies flow cytometry.pdf]
