# Supplementary material for: Comparison of Sources and Methods for the Isolation of Equine Adipose Tissue-Derived Stromal/Stem Cells and Preliminary Results on Their Reaction to Incubation with 5-Azacytidine
Source: Animals (Basel). 2022 Aug 11;12(16):2049. doi: 10.3390/ani12162049 (PMC9404420; doi:10.3390/ani12162049)
Supplement: Supplementary file 1 [file animals-12-02049-s001.zip › Supplementary Table S1_5AZA equine ASC_Study collective.pdf]

Supplementary Table S1. Study collective.

Animal number	Breed	Age	Sex	Type of death	Medical diagnosis (if known)
Horse 1	Mini Shetland Pony	5 years	gelding	euthanasia	colic
Horse 2	Pura Raza Española	1 day	mare	euthanasia	neonatal + orthopedic disease
Horse 3	American Quarter Horse	5 years	gelding	euthanasia	orthopedic + neurologic disease
Horse 4	Fjord Horse	25 years	gelding	slaughter	-
Horse 5	Icelandic Horse	23 years	gelding	euthanasia	colic
Horse 6	Warmblood	5 years	stallion	slaughter	-
Horse 7	German Riding Pony	5 years	stallion	euthanasia	orthopedic disease
Horse 8	American Quarter Horse	26 years	gelding	slaughter	-
Horse 9	American Quarter Horse	14 years	mare	slaughter	-
Horse 10	Warmblood	3 months	mare	euthanasia	orthopedic disease
Horse 11	Warmblood	23 years	gelding	euthanasia	tumor disease
Horse 12	Shire Horse	16 years	gelding	euthanasia	colic
Horse 13	Warmblood	13 years	mare	euthanasia	colic
Horse 14	Warmblood	14 years	gelding	euthanasia	viral infection disease
Horse 15	Shetland Pony	23 years	gelding	euthanasia	colic
Horse 16	Arabian-Mix	12 years	gelding	euthanasia	orthopedic disease
