# Supplementary material for: Comparison of Sources and Methods for the Isolation of Equine Adipose Tissue-Derived Stromal/Stem Cells and Preliminary Results on Their Reaction to Incubation with 5-Azacytidine
Source: Animals (Basel). 2022 Aug 11;12(16):2049. doi: 10.3390/ani12162049 (PMC9404420; doi:10.3390/ani12162049)
Supplement: Supplementary file 1 [file animals-12-02049-s001.zip › Supplementary Table S2_RT-qPCR results Ct mean values.pdf]

**Supplementary Table S2: Numeric results of the real time quantitative PCR before and after exposition to 5-azacytidine.** The experiments were performed with 5 donor horses in two independent repetitions. **(A)** Ct values were measured in triplets and shown as mean ± standard deviation. **(B)** Parameter estimates (with robust standard errors) are demonstrated for OCT4/POU5F1 and MYC. For these two pluripotency markers an one-way ANOVA has been performed, as Ct mean values from T0, T3_nc, T3_ind were more than 4 cycles away from the mean values of NRT and H20. Afterwards, Tukey-HSD (OCT4/POU5F1) and Hochberg (MYC) post hoc tests were performed. **Abbreviations:** T0: day 0, T3: after 3 weeks, nc: negative control, ind: 5-azacytidine induction, p: positive control, NRT: non-reverse transcriptase, IRC: internal run control, GAPDH: glyceraldehyde 3 phosphate dehydrogenase.

**(A)**

| Treatment | OCT4/POU5F1 | MYC | GATA4 | NKX2.5 | TNNI3 | MYH6 | MYH7 | MYF6 |
|---|---|---|---|---|---|---|---|---|
| T0 | 28,26 ± 0,97 | 21,86 ± 0,35 | 32,52 ± 1,15 | 37,22 ± 0,71 | 30,83 ± 0,80 | 36,58 ± 1,49 | 34,95 ± 1,29 | 33,40 ± 1,57 |
| T3-nc | 29,74 ± 0,97 | 22,39 ± 0,62 | 34,14 ± 2,02 | 37,01 ± 0,94 | 32,59 ± 1,23 | 33,55 ± 0,49 | 35,53 ± 1,53 | 34,21 ± 1,60 |
| T3-ind | 29,59 ± 2,00 | 22,59 ± 1,07 | 33,59 ± 1,90 | 36,02 ± 1,91 | 32,32 ± 2,05 | 35,20 ± 1,57 | 34,39 ± 1,54 | 33,03 ± 2,31 |
| p | 28,53 ± 0,50 | 21,70 ± 0,30 | 26,83 ± 0,27 | 30,50 ± 0,15 | 16,28 ± 0,36 | 18,03 ± 0,20 | 18,65 ± 0,53 | 20,85 ± 0,45 |
| NRT | 34,85 ± 2,34 | 33,85 ± 1,71 | 36,70 ± 1,00 | 35,30 ± 0,00 | 32,23 ± 0,59 | 35,10 ± 3,53 | 34,70 ± 0,46 | 38,00 ± 4,11 |
| $H_20$ | 33,73 ± 0,90 | 33,77 ± 1,40 | 32,58 ± 2,15 | 37,30 ± 1,27 | 34,65 ± 1,50 | 37,80 ± 1,75 | 35,45 ± 0,83 | 34,55 ± 1,99 |
| IRC-GAPDH | 17,25 ± 0,16 | 17,47 ± 0,27 | 17,50 ± 0,30 | 17,42 ± 0,47 | 17,43 ± 0,26 | 17,32 ± 0,28 | 17,48 ± 0,17 | 17,25 ± 0,12 |

**(B)**

| MYC | | | | |
|---|---|---|---|---|
| **parameter estimates with robust standard errors:** dependent variable: MYC (Ct value) | | | | |
| | **regression** | | **95% confidence interval** | |
| **parameter** | **coefficient B** | **p value** | **lower limit** | **upper limit** |
| constant term | 22,133 | 0 | 21,556 | 22,711 |
| [treatment T0] | -0,373 | 0,218 | -0,971 | 0,224 |
| [treatment T3-nc] | 0,107 | 0,76 | -0,586 | 0,799 |
| [treatment T3_ind] | 0 | . | . | . |
| [exp.trial=1] | 0,92 | 0,015 | 0,182 | 1,658 |
| [exp.trial =2] | 0 | . | . | . |
| [treatment T0] * [exp.trial=1] | -0,72 | 0,07 | -1,5 | 0,06 |
| [treatment T0] * [exp.trial=2] | 0 | . | . | . |
| [treatment T3-nc] * [exp.trial=1] | -0,613 | 0,163 | -1,481 | 0,254 |
| [treatment T3-nc] * [exp.trial=2] | 0 | . | . | . |
| [treatment T3_ind] * [exp.trial=1] | 0 | . | . | . |
| [treatment T3_ind] * [exp.trial=2] | 0 | . | . | . |
| **R square** = 0,283 (**adjusted R square** = 0,240) | | | | |

| OCT4/POU5F1 | | | | |
|---|---|---|---|---|
| **parameter estimates: dependent variable: OCT4/POU5F1 (Ct value)** | | | | |
| | **regression** | | **95% confidence interval** | |
| **parameter** | **coefficient B** | **p value** | **lower limit** | **upper limit** |
| constant term | 30,067 | 0 | 29,364 | 30,77 |
| [treatment T0] | -1,66 | 0,001 | -2,654 | -0,666 |
| [treatment T3-nc] | -0,78 | 0,123 | -1,774 | 0,214 |
| [treatment T3_ind] | 0 | . | . | . |
| [exp.trial=1] | -0,953 | 0,06 | -1,948 | 0,041 |
| [exp.trial =2] | 0 | . | . | . |
| [treatment T0] * [exp.trial=1] | 0,653 | 0,358 | -0,753 | 2,06 |
| [treatment T0] * [exp.trial=2] | 0 | . | . | . |
| [treatment T3-nc] * [exp.trial=1] | 1,86 | 0,01 | 0,454 | 3,266 |
| [treatment T3-nc] * [exp.trial=2] | 0 | . | . | . |
| [treatment T3_ind] * [exp.trial=1] | 0 | . | . | . |
| [treatment T3_ind] * [exp.trial=2] | 0 | . | . | . |
| **R square** = 0,254 (**adjusted R square** = 0,210) | | | | |
